# Dog as an Outgroup to Human and Mouse

**DOI:** 10.1371/journal.pcbi.0030074

**Published:** 2007-04-27

**Authors:** Gerton Lunter

In a recent contribution to *PLoS Computational Biology,* Cannarozzi, Schneider, and Gonnet published evidence that rodents form an outgroup to human and dog [1], in disagreement with several recent studies suggesting that the dog is an outgroup to the primate–rodent clade [2,3]. The authors' arguments rest on a variety of analyses of human, mouse, and dog genes, using opossum to root the phylogeny. Here I argue that despite the large number of characters used in this study, their results may well be erroneous. I then provide new and, I believe, conclusive evidence in favour of the current consensus phylogeny, and I briefly review other recent studies that support this conclusion.

The problem of determining the evolutionary relationship between all extant mammals has a long history. Traditionally, morphological features were used to group “like” mammals together in a tree, purportedly reflecting their phylogeny. More recently, molecular data have generally confirmed these inferences, but have also led to surprising revisions. While sequence analysis is more objective than morphology, it nevertheless emerged that it has its own set of issues, and some phylogenies remain contentious. In [1], Cannarozzi et al. suggested that this contention extends to the phylogeny of human, mouse, and dog, and inferred a phylogeny of these species that disagrees with a recently emerging consensus. Here I challenge their findings, providing new evidence in support of the consensus phylogeny, and suggest that their results may have been biased by long branch attraction (LBA), a known issue in molecular phylogenetic inference.

It is well-known that phylogenetic inferences can be biased, and may be inaccurate even with strong bootstrap or posterior support. Felsenstein showed that in parsimony analyses, long branches in the phylogeny tend to attract one another [4]. In contrast to what the authors claim, maximum likelihood methods, although less vulnerable, are similarly affected by LBA [5], particularly when small numbers of taxa are used [6]. This methodological bias has led to various erroneous inferences, such as the now-discredited claim that “the guinea pig is not a rodent” [7,8]. Perhaps counterintuitively, the effect of LBA does not diminish with increasing amounts of sequence data. To quote from a review, “spurious conclusions are often derived from an over-credibility of enormous numbers of nucleotide or amino acid characters (e.g., complete genomes) when combined with poor taxon sampling” [9].

The recently emerging consensus on mammalian phylogeny based on molecular data is surprisingly different from the traditional, morphological phylogeny [2,3]. It proposes four mammalian cohorts, including the Laurasiatheria (of which the dog lineage is part), which separated from the Euarchontoglires about 85–95 million years ago (Mya) [10]. The subsequent speciation separating the Euarchontoglires into Glires (including rodents) and Euarchonta (which includes primates) occurred roughly 80 Mya. The difference is small compared with the total branch length to opossum (180 + 90 My), so that a relatively small bias would suffice to bring about a topology change. As the mouse genome sequence has been evolving fast relative to those of human and dog [11], its branch is expected to be affected by LBA to the opossum branch, which would result in the reported grouping.

These considerations throw some doubt on both the parsimony and maximum likelihood analyses. What about the genome rearrangement argument? After all, genome rearrangements are large-scale but relatively infrequent events, so that the parsimony approximation might be justified. However, the opossum genome had not yet been assembled, and the authors had to resort to chicken, which diverged ∼310 Mya from the mammalian lineage, considerably earlier than the opossum did. Moreover, there is strong evidence for hotspots of breakage [12] and breakpoint reuse [13], discounting the “random breakage” model. The use of (nuclear) gene orderings to analyze rearrangements further exacerbates these issues, as it affords little power to resolve breakpoints and artificially increases inhomogeneities in breakage rates, because of large and highly variable intergenic distances. For these reasons, the parsimony approximation may well be invalid, which makes LBA a concern for the genome rearrangement analysis, too.

I thus considered whether the reported tree might be incorrect. To investigate the issue, I used a simple (and, to my knowledge, novel) summary statistic based on the distribution of transposable elements (TEs) in pairwise alignments, which does not require an outgroup genome to root the phylogeny. If a family of TEs is specific to lineage *x* when compared with *y*, each occurrence in *x* is expected to be located opposite a gap in a whole-genome alignment of species *x* to *y*. In contrast, if the family is ancestral to *x* and *y,* a proportion of TEs will have survived in both species and will align. To quantify the evidence for these alternatives, I defined a statistic A(*y*|*x*) (for “ancestralness”) as the proportion of nucleotides from a particular TE family in species *x* that is aligned to a secondary species *y*. This statistic is near-zero if a family of TEs is specific to *x*, and non-zero if it is ancestral to the species split. For an outgroup *x* and a particular family of TEs, the statistics A(*y*|*x*) are thus expected to be consistent across ingroup species *y* (either zero, or non-zero, for all). In contrast, for an ingroup species, some TE families may be ancestral with respect to another ingroup, but lineage-specific when compared with the outgroup. Provided such TE families exist, this would then determine the topology of the phylogeny.

The results (Figure 1 and Table 1) show clear support for the rodent–primate grouping. For example, the MLT2B2 long terminal repeat element is clearly ancestral in the human-to-mouse and mouse-to-human comparisons (A > 0.20), but is highly lineage-specific in the other comparisons, each of which include the dog (A < 0.03 for all). This pattern can be explained if dog is assumed to be an outgroup to both human and mouse, and that the element has been active primarily between the two speciation events. The same pattern was observed for several other TE families (MLT1A0, MLT2B1, L1MA9, L1MB1, L1MC1, MER31A, MER21B, MER34), while no examples supporting alternative groupings were found. Unlike analyses based on nucleotide characters, TE-based studies are not expected to suffer from LBA, because the size of TEs allows for reliable homology assignments (if well-anchored alignments are used), and the marked differences between the TE insertion and small deletion processes means that back mutations are rare. It thus appears that the dog lineage is basal to the primate and rodent lineages.

**Figure 1:**
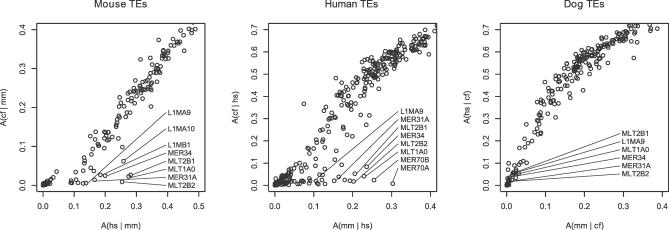
Evidence for the ((Human, Mouse), Dog) Phylogeny Shown are the ancestralness A(*y|x*) for a range of TEs families in a species *x* (mouse, mm; human, hs; dog, cf), compared with the two remaining auxiliary species *y*. Data are shown for all TEs that were present in at least 500 copies covering 50 kb or more in species *x*. When *x* is the outgroup, the ancestralness is expected to be consistent across auxiliary ingroup species *y,* while for ingroup species, some TE families may be ancestral (A > 0) for the second ingroup, but lineage-specific (A ≈ 0) for the outgroup. All three scatter plots support dog as the outgroup species.

**Table 1:**
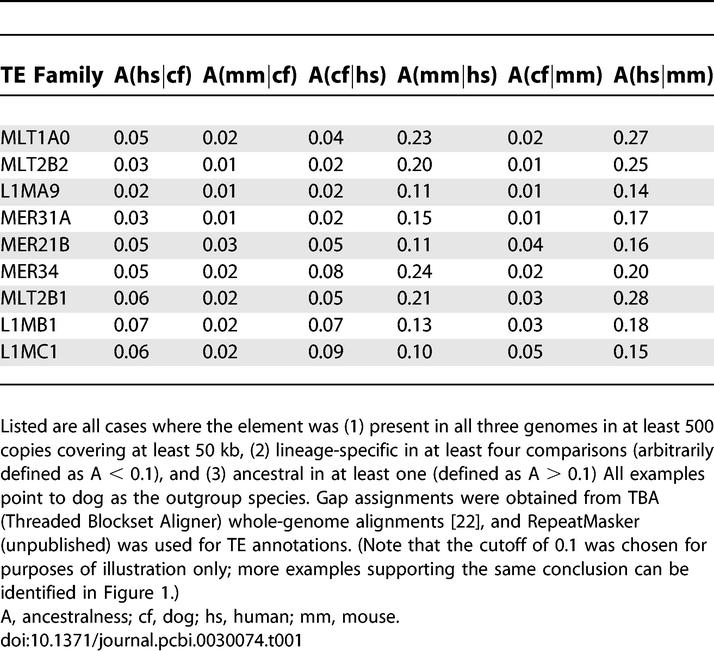
Ancestralness of TE Families in the Six Pairwise Comparisons between Human, Dog, and Mouse

Numerous recent studies support this conclusion. When many taxa are analyzed simultaneously, the dog consistently appears as an outgroup to human and mouse, when using either nuclear or mitochondrial DNA [2,3,9,14–16]. Studies of rare genomic changes (which are less vulnerable to LBA) consistently support this grouping. For example, by rooting the phylogeny using the consensus sequence of TEs, the evolutionary distance between the speciation events was estimated to be 0.024 substitutions per site [11]. In another study, two of the TE families found here, MLT1A0 and L1MA9, were identified as clear examples supporting the rodent–primate grouping [17], and a recent analysis of several single TE insertions provides additional support [18], as does a method that uses multiple alignments of TEs to infer phylogenies in very similar ways to ours [19]. Rare indels at homologous positions in otherwise well-conserved protein-coding genes also support this phylogeny [20]. Finally, a large cluster of PRAME genes that is absent in chicken and dog, but present in homologous locations in human and mouse, again support the same grouping [21].

Taken together with the possible influence of LBA on the analysis of Cannarozzi et al. [1], it appears unjustified to continue to consider the phylogeny of primates, rodents, and canines as contentious. 

## Abbreviations

LBA: long branch attraction
Mya: million years ago
TE: transposable element
